# ER-α36 Promotes the Malignant Progression of Cervical Cancer Mediated by Estrogen *via* HMGA2

**DOI:** 10.3389/fonc.2021.712849

**Published:** 2021-07-14

**Authors:** Chunyan Wang, Tianli Zhang, Kun Wang, Shuo Zhang, Qing Sun, Xingsheng Yang

**Affiliations:** ^1^ Department of Obstetrics and Gynecology, Qilu Hospital, Shandong University, Jinan, China; ^2^ School of Medicine, Cheeloo College of Medicine, Shandong University, Jinan, China

**Keywords:** cervical cancer, ER-α36, HMGA2, proliferation, metastasis

## Abstract

**Objectives:**

Estrogen is proven to promote the malignant behaviors of many cancers *via* its receptors. Estrogen receptor alfa 36 (ER-α36) is a newly identified isoform of estrogen receptor alfa (ER-α), the role of ER-α36 in regulating the effects of estrogen and its potential impact on human cervical cancer is poorly understood.

**Methods:**

Immunohistochemistry staining was used to evaluate the expression of ER-α36, estrogen receptor alfa 66 (ER-α66) and their prognostic values in cervical cancer. The effects of ER-α36 and ER-α66 on the proliferation and metastasis of cervical cancer were measured *in vitro*. A xenograft tumor assay was used to study the tumorigenesis role of ER-α36 *in vivo*. Furthermore, the functional gene at the downstream of ER-α36 was obtained *via* next-generation sequencing, and the biological functions of high mobility group A2 (HMGA2) in cervical cancer cells were investigated *in vitro*.

**Results:**

ER-α36 was over-expressed in cervical cancer tissues and elevated ER-α36 expression was associated with poor prognosis in cervical cancer patients. High expression of ER-α36 promoted the proliferation, invasion and metastasis of cervical cancer cells mediated by estrogen, while silencing ER-α36 had the opposite effects. Further research showed that HMGA2 was a downstream target of ER-α36 in cervical cancer cells. The oncogenic effect of ER-α36 was attenuated after HMGA2 knockdown.

**Conclusions:**

High expression of ER-α36 was correlated with a poor prognosis in cervical cancer by regulating HMGA2. ER-α36 could be a prognostic biomarker and a target for cervical cancer treatment.

## Introduction

Cervical cancer (CC) is the fourth leading cause of cancer death in women. According to the latest statistics, there were 604127 incident cases and 341831 deaths due to CC worldwide in 2020 (1). Squamous cell Carcinoma (SCC) and adenocarcinoma (AC) are the most common histological subtypes accounting for about 70% and 25% of all cervical cancers, respectively (2). At present, radical surgery is the first‐ line treatment for early‐stage CC, the combination of γ- irradiation and cisplatin-based chemotherapy is the standard treatment for advanced CC (3). Although great progress has been made in disease prevention with the emergence of HPV vaccination and early screening, the incidence and mortality of CC are still high in low and middle-income countries (4). Therefore, it is important to elucidate the potential oncogenic molecular mechanisms in CC and develop new therapy methods.

Human papillomavirus (HPV) is a necessary but not sufficient cause of CC (5). In human cervical squamous epithelium, most lesions containing high-risk HPVs do not progress to CC, implicating other factors may be involved in the genesis of CC. Long-term use of oral contraceptives and high parity increase the risk of CC in woman with HPV infection (6, 7). Exposure to diethylstilbestrol is associated with increased risk of cervical high-grade squamous neoplasia (8). Chronic estrogen treatment induced the genesis of cervical squamous cancer in HPV type 16 transgenic mice (9). These studies suggest that estrogen is an etiological cofactor for CC.

Estrogen influences physiological and pathological processes in various tissues through its receptors. ER-α is the major estrogen receptor expressed in the cervix. Researchers found that ER-α plays a critical role in cervical carcinogenesis in transgenic mice (10). ER-α is the product of ESR1 gene. The ESR1 gene not only serves as a template for a full-length 66 kDa protein(ER-α66), but also for two alternative isoforms(ER-α46 and ER-α36) (11). ER-α36 lacks transcriptional activation domains (AF-1 and AF-2), but retains dimerization, DNA-binding and partial ligand-binding domains. ER-α36 is mainly located in plasma membrane and cytoplasm, and mediates rapid estrogen signaling pathways (11–15). Previous studies have indicated that high expression of ER-α36 is associated with a more aggressive phenotype in various cancers, including breast cancer (16–18), endometrial cancer (14), gastric cancer (19), lung adenocarcinoma (20) and laryngeal cancer (21). However, the role of ER-α36 in regulating the effect of estrogen and its potential impact on human CC remains unknown.

In this study, we first verified that ER-α36 was remarkably over-expressed in CC tissues and was associated with poor prognosis of CC. Through *in vitro* and vivo experiments, we demonstrated that ER-α36 promoted the malignant progression of CC mediated by estrogen, and these effects on biological behavior were achieved by regulating the expression of HMGA2. To reveal the correlations between ER-α36 and HMGA2, we further researched the expression, biological functions and clinical significance of HMGA2 in CC.

## Materials and Methods

### Human CC Specimens

A total of 117 cases of paraffin‐embedded primary CC samples, 30 cases of paraffin‐embedded cervical intraepithelial neoplasia (CIN) and 60 cases of paraffin-embedded normal cervix samples were collected from the Department of Pathology, Qilu Hospital, from January 2012 to December 2014. In addition, 12 cases of CC tissues were collected from patients with CC who underwent primary surgery in Qilu Hospital, while the 8 normal cervix tissues were from patients who received hysterectomy due to benign gynecologic tumors. All the paraffin‐embedded samples possessed intact follow‐up information. Written informed consents were obtained from all the patients. Ethical approval was issued by the Ethics Committee of Shandong University Qilu Hospital and the approval number is KYLL-2018-174.

### Immunohistochemistry (IHC)

The 4um-thick paraffin‐embedded tissue sections were dewaxed with xylene and rehydrated in an ethanol gradient. After antigen retrieval, these sections were incubated with a primary antibody at 4°C overnight, then were incubated with secondary antibodies for 30 minutes at room temperature. Subsequently, these slides were stained with 3,3’-diaminobenzidine detection system and haematoxylin.

These sections were scored by two trained pathologists according to the extent and intensity of staining. The percentage of positively stained cells was scored as (0, 0%; 1, 1%–25%; 2, 25%–50%; 3, 50%–75%; 4, 75%–100%) and the intensity of staining was scored as (0, negative; 1, weak; 2, moderate; 3, strong) (22). The final score was the product of the two scores multiplied together. The final score less than 4 indicated low expression and greater than 5 indicated high expression.

### Quantitative Reverse‐Transcription Polymerase Chain Reaction (qRT‐PCR)

Total RNA was extracted by TRIzol reagent (15596018; Invitrogen). Complementary DNA (cDNA) was synthesized by the PrimeScript RT reagent Kit (FSQ‐301; Toyobo Biotech Co Ltd, OSAKA, Japan). Real‐time PCR was performed using the SYBR Green Real‐time PCR Master Mix (QPK‐201; Toyobo Biotech Co Ltd, OSAKA, Japan). The message RNA(mRNA) level of specific genes was normalized against glyceraldehyde 3‐phosphate dehydrogenase (GAPDH) using the comparative Ct method (2^−ΔΔCt^). The primers used are shown in the **Supplementary Table S1**.

### Cell Lines and Cell Culture

H8, siha, hela, caski and C33a cell lines were obtained from the Key Laboratory of Gynecologic Oncology of Shandong Province. Cells were cultured in phenol-red-free Roswell Park Memorial Institute (RPMI) 1640 (Gibco, USA) supplemented with 10% fetal bovine serum (FBS) (04-001-1 A, Biological Industries, Kibbutz Beit‐Haemek, Israel). All cells were cultured in a humidified incubator at 37°C with 5% CO_2_.

### Cell Transfection and the Production of Stable Cell Lines

Small interfering RNA (siRNA) for ER-α36, ER-α66, HMGA2 and their negative control si-NC were obtained from GenePharma (Shanghai, China). Caski and hela cells at the 50% confluence were transiently transfected with siRNAs using INTERFERin (Polyplus, USA) according to the manufacturer’s protocol. ER-α36 knockdown and overexpression lentiviruses were purchased from Genechem (Shanghai, China). Caski and hela cells were infected with lentivirus (MOI: 20) and selected with 1.5 mg/ml puromycin for about 1 week. The efficiency of knockdown or overexpression was detected by qRT-PCR and western blotting. The siRNA sequences are shown in the supplementary material.

### Western Blotting

Cells and tissues were lysed in RIPA (Beyotime, Shanghai, China) supplemented with PMSF according to the manufacturer’s recommendations. The protein concentration was calculated by the BCA Kit (Beyotime, Shanghai, China). Proteins were separated by SDS-PAGE electrophoresis, then were transferred to PVDF membrane. After blocking in the 5% milk for 1 hour at room temperature, these membranes were incubated with primary antibodies overnight at 4°C. Subsequently, these membranes were labeled with the corresponding HRP-conjugated secondary antibodies (Cell Signaling Technology, Danvers) for 1 hour at room temperature. Protein bands were detected by enhanced chemiluminescence detection kit (ECL ORT2655, PerkinElmer, Waltham, MA, USA). ImageJ 1.47 was used to analyze the relative protein level. GAPDH was used as an endogenous control.

### Antibodies and Reagents

Antibodies for ER-α66(ab108398) and Ki67(ab16667) were purchased from Abcam (Cambridge, UK). ER-α36 (SAB1306666) antibody for western blotting was purchased from Sigma-Aldrich (St. Louis, MO, USA), ER-α36(bs-23769R) antibody for IHC was purchased from Bioss (Bioss, China). The antibody for HMGA2 (20795-1-AP) was purchase from Proteintech (Proteintech Group, USA) and the antibody for GAPDH (GB11002) was purchased from Servicebio (Servicebio, China). 17β-estradiol (E_2_) (E8875) (dissolved by ethanol) was purchased from Sigma (St. Louis, USA) and 17β-estradiol pellets (SE-121) were purchased from Innovative Research (IRA, USA).

### Cell Viability Assay

Cells (10^3^ per well) were seeded into 96-well plates and cultured for 1–6 days, Cell Counting Kit-8 (CCK8) (APExBIO, #K1018) was used to detect cell viability following the manufacturer’s protocol. The absorbance at 450 nm was detected by a microtiter plate reader (Thermo Scientific).

### Colony Formation Assay

Cells (10^3^ per well) were seeded into 6-well plates and cultured for 10–14 days. The colonies were fixed by methanol and stained by 0.5% crystal violet. Colonies more than 50 cells were counted.

### Cell Migration and Invasion Assay

Migration and invasion assays were performed in transwell chambers (8-μm pores, BD Biosciences, USA) inserted in 24-well plates without or with Matrigel (356234, Corning Incorporate, NY). Cells (10^5^ per well) were seeded into the upper chambers in serum-free medium, medium supplemented with 20% FBS were added to the lower chambers. After cultured for an appropriate time in an incubator, cells that have migrated through the membrane were fixed with methanol, stained with 0.5% crystal violet and observed under a light microscope.

### Cell Cycle Assessment

Cells were collected and stained with PI/RNase Staining Buffer (550825, BD Bioscience, Franklin Lakes, NJ, USA) following the manufacturer’s protocol. Flow cytometry was used to evaluate the distribution of cell cycle.

### High-Throughput Differential Gene Expression Analysis

The high-throughput RNA-seq experiments were conducted by Annoroad (Beijing, China). In brief, caski cells were transfected with si-ER-α36 or NC (n = 3) for 48 h and treated with 1nM E_2_ for 24h. The RNA preparation and library preparation for transcriptome sequencing were performed according to the manufacturer’s instructions. Fragments per kilobase of transcript per million fragments mapped (FPKM) was used to estimate gene expression levels. The differentially expressed mRNAs were selected with fold change > 2 or fold change < 0.5 and p value < 0.05 by R package edgeR or DESeq2.

### Animal Experiment

The animal study was reviewed and approved by the Ethics Committee of Shandong University Cheeloo College of Medicine, the approval number is 21002. Female BALB/c nude mice (4-6 weeks old; NBRI of Nanjing University, Nanjing, China) were implanted subcutaneously with 0.36 mg of 60-day release 17β-estradiol pellets (Innovative Research, TX). Caski cells transfected with shNC, shER-α36, NC-LV and NC-ER-α36 were collected, 5 × 10^6^ cells were subcutaneously injected into the axilla of each mouse. The tumor size was measured by Vernier calliper once every 2 days, and tumor volumes were calculated using the equation: length × width^2^ × 0.5. Three weeks post-injection, these mice were sacrificed and tumors were removed, photographed and weighed.

### Statistical Analysis

Each assay was repeated at least 3 times independently. The data was expressed as the means ± SEM. Student’s t test was applied to compare two independent groups and One-way ANOVA was used to compare multiple groups. The correlation among ER-α36, ER-α66 and clinicopathological characteristics were assessed by chi-square (χ2) or Fisher test. Overall survival analysis was performed by Kaplan‐Meier method with log‐rank test. Survival data was evaluated by univariate and multivariate Cox regression analyses to evaluate the independent factors of patients’ outcomes. All the analyses above were performed by SPSS v22.0 (SPSS, Inc., Chicago, IL, USA). Images were processed by GraphPad Prism 8.00 (GraphPad Software, La Jolla, CA, USA) and Adobe Photoshop CC 2019 (Adobe, San Jose, CA, USA). Differences were considered statistically significant when P < 0.05(*p < 0.05, **p < 0.01, ***p < 0.001).

## Results

### Expression and Prognostic Significance of ER-α36 and ER-α66 in CC

**Figure 1:**
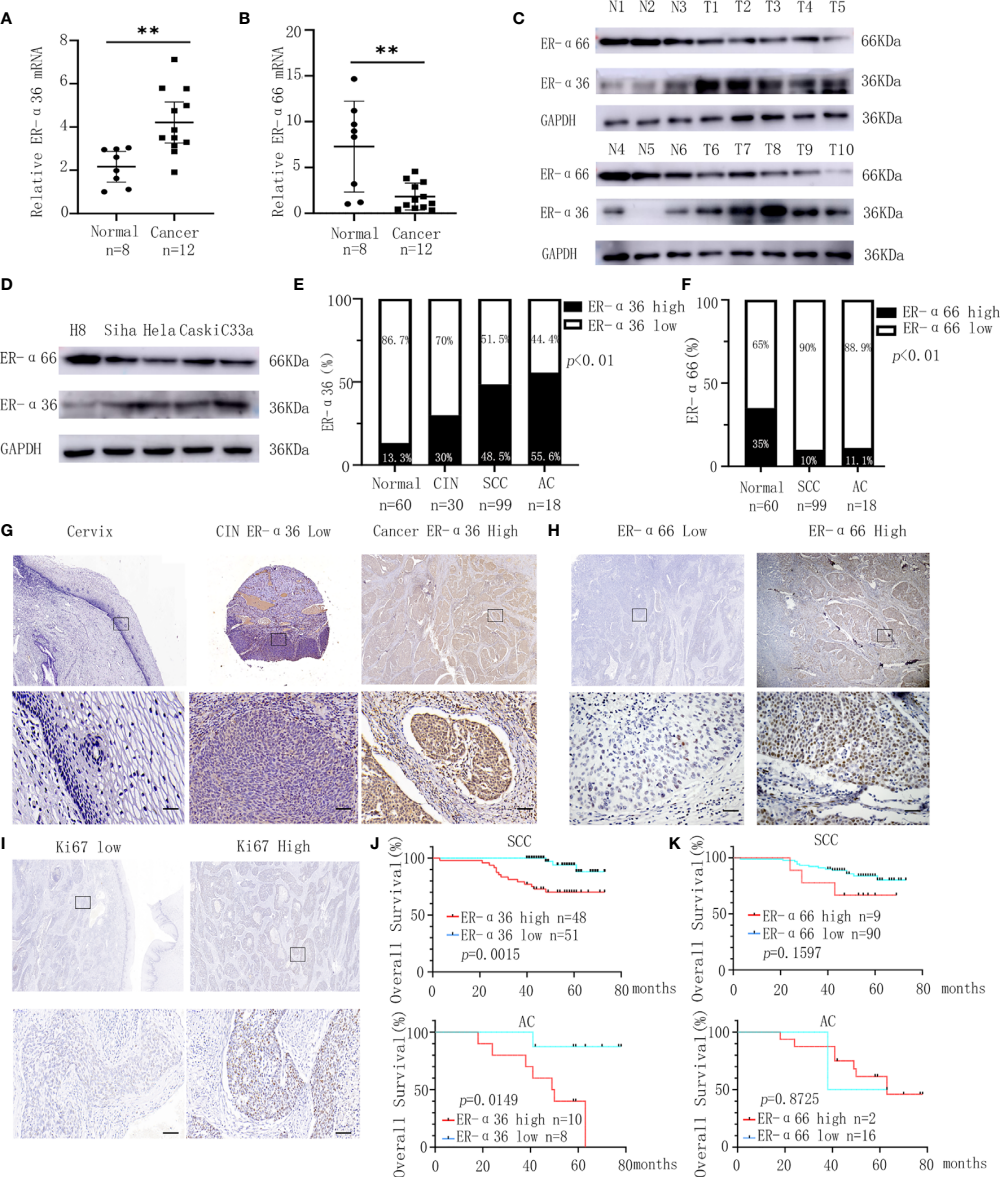
Expression and prognostic significance of ER-α36 and ER-α66 in CC. **(A, B)** The mRNA levels of ER-α36 and ER-α66 in CC tissues and normal cervical tissues. **(C)** The protein levels of ER-α36 and ER-α66 in CC tissues (T1-T10) and normal cervical tissues(N1-N6). **(D)** The protein levels of ER-α36 and ER-α66 in H8, siha, hela, caski and C33a cell lines. **(E)** The high and low expression rate of ER-α36 in 60 cervical tissues, 30 CIN tissues, 99 cervical SCC tissues and 18 cervical AC tissues. **(F)** The high and low expression rate of ER-α66 in 60 cervical tissues, 99 cervical SCC tissues and 18 cervical AC tissues. **(G)** Representative images of IHC staining of ER-α36 in CC tissues, CIN and cervical tissues (upper, ×40; lower, ×400, Scale bar: 50 μm). **(H, I)** Representative images of IHC staining of ER-α66 and Ki67 in CC tissues (upper, ×40; lower, ×400, Scale bar: 50 μm). **(J)** High expression of ER-α36 was significantly correlated with low overall survival rate in cervical SCC and cervical AC. **(K)** The expression of ER-α66 displayed no prognostic significance in cervical SCC and cervical AC. (**p < 0.01).

We detected the expressions of ER-α36 and ER-α66 in CC tissues and cervix tissues with qRT-PCR and western blotting, the results showed that the mRNA and protein levels of ER-α36 were higher in cancer tissues, while the mRNA and protein levels of ER-α66 were higher in cervix tissues (**Figures 1A–C**).Then we detected their expressions in H8,siha,hela,caski and C33a cell lines, western blotting demonstrated that ER-a36 was significantly over-expressed in four CC cell lines (siha,hela,caski and C33a) compared with H8, the normal cervical epithelial cells, while ER-α66 was over-expressed in H8 cells (**Figure 1D**).These results indicated the possible roles of ER-α36 and ER-α66 in CC.

For further investigation, we carried out IHC staining to analyze the expression of ER-a36 and ER-a66 in 60 cases of normal cervix tissues and 117 cases of CC tissues including 99 cases of cervical SCC and 18 cases of cervical AC. Positive ER-α36 staining was mainly detected in cellular membrane and cytoplasm in CC tissues, while positive ER-α66 staining was mainly detected in the nucleus. These patients were divided into high expression groups and low expression groups based on the IHC scores (**Figures 1G–I**). The expression of ER-α36 was significantly higher in cervical SCC (48.5%, 48/99 cases) and AC (55.6%, 10/18 cases) tissues than in CIN tissues (30%, 9/30 cases) and cervix tissues (13.3%, 8/60 cases) (**Figure 1E**). However, the expression of ER-α66 was lower in cervical SCC (10%, 9/90 cases) and AC (11.1%, 2/18 cases) tissues than in cervix tissues (35%, 21/60 cases) (**Figure 1F**). Kaplan–Meier survival curves showed that high ER-α36 expression was remarkably associated with unfavorable prognosis in cervical SCC and AC patients (**Figure 1J**), while the expression of ER-α66 displayed no prognostic significance in CC patients (**Figure 1K**). Collectively, ER-α36 was up-regulated and correlated with the poor prognosis in CC.

### Clinical Significance of ER-α36 and ER-α66 in CC

To assess the clinical significance of ER-α36 and ER-α66, we analyzed the correlation among ER-α36, ER-α66 and the clinicopathological features of cervical SCC and AC patients. In patients with cervical SCC, high expression of ER-α36 was significantly associated with advanced International Federation of Gynecology and Obstetrics (FIGO) stage (p=0.026), deeper stromal invasion (p=0.032), positive lymph node metastasis (p=0.005) and high expression of Ki67 (p<0.001), whereas there is no correlation between ER-a66 and these features (**Table 1**).In cervical AC patients, elevated ER-α36 was associated with positive lymph node metastasis (p=0.036), however no correlation was observed between ER-a66 and this feature (**Table 2**).

**Table 1:** Association between ER-α36/ER-α66 expression and clinicopathologic characteristics of cervical SCC patients.

Characteristic	Cases	ER-α36 expression		p-value	ER-α66 expression		p-value
		Low	High		Low	High	
Age				0.108			0.495
≤50	60	27	33		56	4	
>50	39	24	15		34	5	
Menopausal status				0.639			0.152
Postmenopausal	29	16	13		24	5	
Premenopausal	70	35	35		66	4	
Differentiation				0.379			0.320
Poor	68	33	35		60	8	
Well/moderate	31	18	13		30	1	
FIGO stage				0.026*			1.000
I	81	46	35		74	7	
II	18	5	13		16	2	
Tumor Size				0.482			0.459
<4cm	71	35	36		66	5	
≥4cm	28	16	12		24	4	
DSI				0.032*			1
<1/2	9	8	1		9	0	
≥1/2	90	43	47		81	9	
LNM				0.005**			0.053
Negative	67	41	26		64	3	
Positive	32	10	22		26	6	
LVSI				0.879			0.553
Negative	79	41	38		73	6	
Positive	20	10	10		17	3	
HPV				1			0.440
Negative	2	1	1		1	1	
Positive	73	30	43		67	6	
Ki-67 status				<0.001***			0.773
Low	56	39	17		50	6	
High	43	12	31		40	3	

SCC, squamous cell carcinoma; FIGO, International Federation of Gynecology and Obstetrics; DSI, deep stromal invasion; LNM, lymph node metastasis; LVSI, lymphvascular space involvement; HPV, human papillomavirus.

*p < 0.05, **p < 0.01, ***p < 0.001, χ2 test.

**Table 2:** Association between ER-α36/ER-α66 expression and clinicopathologic characteristics of cervical AC patients.

Characteristic	Cases	ER-α36 expression		p-value	ER-α66 expression		p-value
		Low	High		Low	High	
Age				0.608			1
≤50	13	5	8		11	2	
>50	5	3	2		5	0	
Menopausal status				0.608			1
Postmenopausal	5	3	2		5	0	
Premenopausal	13	5	8		11	2	
Differentiation				1			1
Poor	12	5	7		11	1	
Well/moderate	6	3	3		5	1	
FIGO stage				0.216			1
I	15	8	7		13	2	
II	3	0	3		3	0	
Tumor Size				0.608			1
<4cm	13	5	8		11	2	
≥4cm	5	3	2		5	0	
DSI				0.559			1
<1/2	3	2	1		3	0	
≥1/2	15	6	9		13	2	
LNM				0.036*			0.490
Negative	13	8	5		12	1	
Positive	5	0	5		4	1	
LVSI				1			1
Negative	16	7	9		14	2	
Positive	2	1	1		2	0	
HPV				0.462			1
Negative	1	1	0		1	0	
Positive	12	5	7		11	1	
Ki-67 status				1			0.137
Low	11	5	6		11	0	
High	7	3	4		5	2	

AC, adenocarcinoma; FIGO, International Federation of Gynecology and Obstetrics; DSI, deep stromal invasion; LNM, lymph node metastasis; LVSI, lymphvascular space involvement; HPV, human papillomavirus.

*p < 0.05, Fisher test.

In addition, we performed univariate and multivariate analyses to evaluate the prognostic values of ER-α36, ER-α66 and other clinicopathological factors. In patients with cervical SCC, univariate Cox regression analysis demonstrated advanced FIGO stage (p=0.042), positive lymph node metastasis (p=0.003), and high ER-α36 expression (p=0.005) were all significantly associated with a low overall survival rate. Multivariate Cox regression analysis confirmed that high expression of ER-α36 could be an independent factor to predict poor survival of cervical SCC (p=0.028) together with positive lymph node metastasis (p=0.025, **Table 3**). In cervical AC patients, univariate Cox regression analyses suggested that FIGO stage (p=0.023), lymph node metastasis (p=0.038) and ER-α36 expression (p =0.042) had strong correlations with prognosis (**Table 4**).

**Table 3:** Univariate and multivariate analyses of overall survival in cervical SCC patients.

Variables	Univariate analysis			Multivariate analysis		
	HR	95%CI	P-value	HR	95%CI	P-value
Age	1.138	0.432-2.999	0.794			
≤50 *vs.* >50						
Differentiation	0.424	0.122-1.478	0.178			
oor *vs.* Well/Moderate						
FIGO stage	2.815	1.037-7.640	0.042*	0.588	0.111-0.861	0.310
I *vs.* II						
Tumor	1.809	0.688-4.757	0.229			
<4cm *vs.* ≥4cm						
DSI	0.043	0-61.321	0.395			
<1/2 *vs.* ≥1/2						
LNM	0.1218	0.081-0.591	0.003**	0.309	0.111-0.861	0.025*
Negative *vs.* Positive						
LVSI	0.753	0.245-2.310	0.620			
Negative *vs.* Positive						
HPV	0.047	0-68.56	0.615			
Negative *vs.* positive						
ER-α36 expression	0.169	0.048-0.589	0.005**	0.485	0.254-0.927	0.028*
Low *vs.* High						
ER-α66 expression	0.419	0.120-1.460	0.172			
Low *vs.* High						

SCC, squamous cell carcinoma; HR, hazard ratio; CI, confidence interval; FIGO, International Federation of Gynecology and Obstetrics; DSI, deep stromal invasion; LNM, lymph node metastasis; LVSI, lymphvascular space involvement; HPV, human papillomavirus.

*p < 0.05, **p < 0.01, the univariate and multivariate Cox regression analysis.

**Table 4:** Univariate and multivariate analyses of overall survival in cervical AC patients.

Variables	Univariate analysis			Multivariate analysis		
	HR	95%CI	P-value	HR	95%CI	P-value
Age	0.516	0.122-2.182	0.368			
≤50 *vs.* >50						
Differentiation	2.183	0.433-11.014	0.345			
Poor *vs.* Well/Moderate						
FIGO stage	2.154	0.031-0.774	0.023*	0.163	0.022-1.215	0.077
I *vs.* II						
Tumor	1.255	0.252-6.259	0.781			
<4cm *vs.* ≥4cm						
DSI	1.589	0.195-12.923	0.665			
<1/2 *vs.* ≥1/2						
LNM	0.227	0.056-0.923	0.038*	0.268	0.039-1.842	0.181
Negative *vs.* Positive						
LVSI	1.475	0.172-12.628	0.723			
Negative *vs.* Positive						
HPV	0.043	0-121.674	0.678			
Negative *vs.* Positive						
ER-α36 expression	0.108	0.013-0.921	0.042*	0.362	0.025-5.306	0.458
Low *vs.* High						
ER-α66 expression	0.842	0.101-6.985	0.873			
Low *vs.* High						

AC, adenocarcinoma; HR, hazard ratio; CI, confidence interval; FIGO, International Federation of Gynecology and Obstetrics; DSI, deep stromal invasion; LNM, lymph node metastasis; LVSI, lymphvascular space involvement; HPV, human papillomavirus.

*p < 0.05, the univariate Cox regression analysis.

### ER-α36 Silence Inhibits Proliferation and Metastasis of CC Cells Mediated by E_2_ *In Vitro*

**Figure 2:**
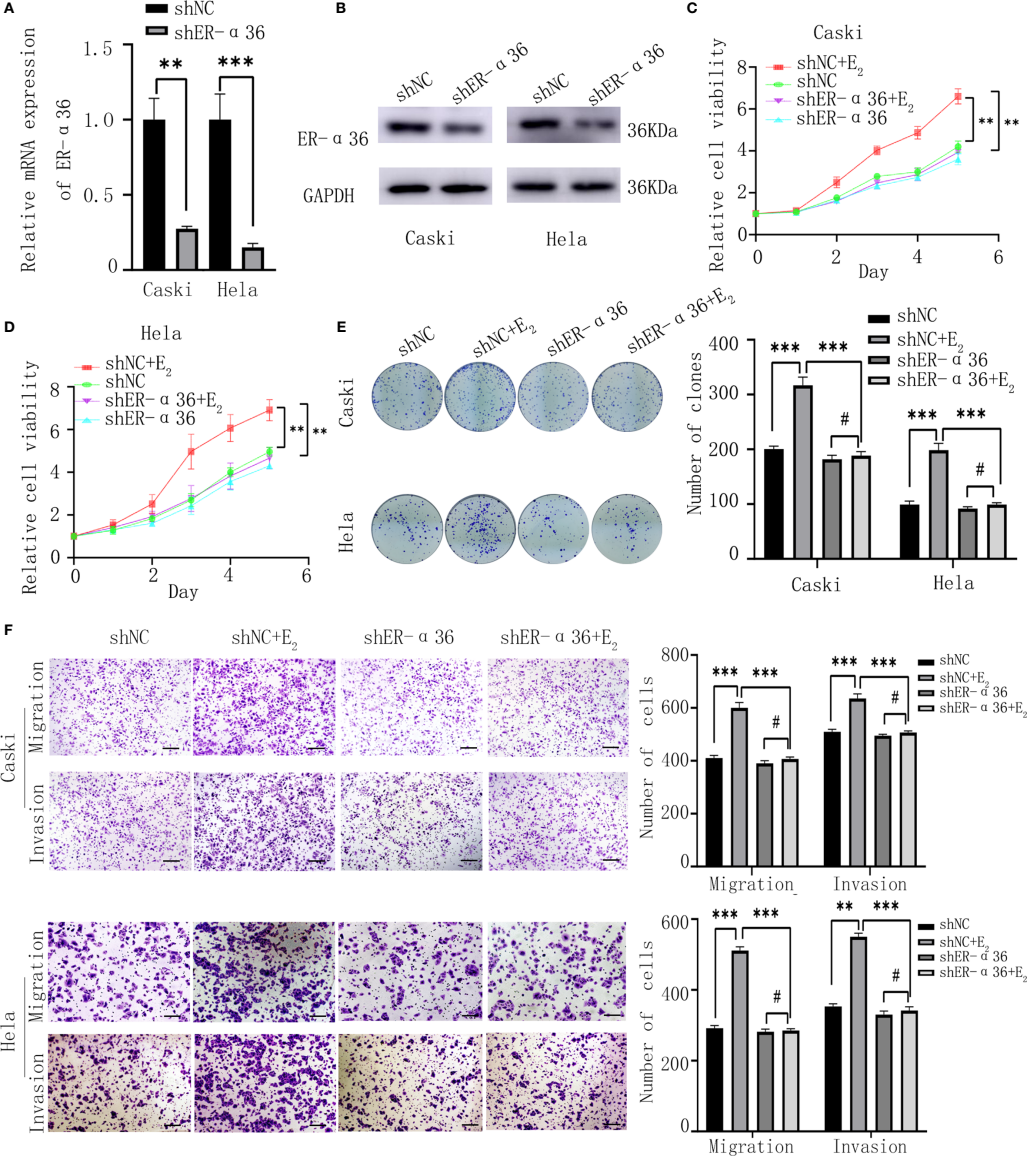
ER-α36 silence inhibits proliferation and metastasis of CC cells mediated by E_2_ in *vitro*. Caski, hela cells were stably transfected with shNC, shER-α36 and treated with ethanol or 1nM E_2_. **(A, B)** qRT‐PCR and western blotting analysis of the mRNA and protein levels of ER-α36 in transfected caski and hela cells. **(C, D)** Proliferation of transfected caski and hela cells was measured by CCK-8 assay. **(E)** Colony formation efficiency of caski and hela cells were assessed by clonogenic assay after ER-α36 knockdown. **(F)** Migration and invasion of caski and hela cells were evaluated by transwell assay after ER-α36 knockdown. Scale bar: 50μm. (Data are mean ± SEM, ^#^p > 0.05, **p < 0.01, ***p < 0.001, n = 3).

Caski and hela cells were stably transfected with shER-α36 and negative control (shNC) (**Figures 2A, B**). Cells were divided into 4 groups; the shNC groups (cells transfected with shNC and treated with ethanol), the shNC+E_2_ groups (cells transfected with shNC and treated with 1nM E_2_), the shER-α36 groups (cells transfected with shER-α36 and treated with ethanol) and shER-α36+E_2_ groups (cells transfected with shER-α36 and treated with 1nM E_2_). The CCK8 assay showed that cells transfected with ShER-α36 (shER-α36 groups vs. ShER-α36+E_2_ groups) exhibited lower sensitivity to E_2_ stimulation than the control groups (shNC groups vs. shNC+E_2_ groups). In the shNC+E_2_ groups, E_2_ promoted the proliferation of CC cells compared to shNC groups. However, there is no significant difference in proliferation between shER-α36 groups and shER-α36+E_2_ groups. In the ShER-α36+E_2_ groups, the proliferation induced by E_2_ was suppressed in comparison to the shNC+E_2_ group. Nevertheless, there was no remarkable difference in cell viability between shNC and shER-α36 groups (**Figures 2C, D**). The clonogenic assay also confirmed these results (**Figure 2E**). In addition, we transfected si-ER-α66 and negative control (si-NC) into caski and hela cells (**Supplementary Figures 1A, B**), and there was no significant difference in the capability of proliferation between si-NC+E_2_ groups (cells transfected with si-NC and treated with 1nM E_2_) and si-ER-α66+E_2_ groups (cells transfected with si-ER-α66 and treated with 1nM E_2_) (**Supplementaryl Figures 1C–E**).

Considering that the expression of ER-α36 was associated with lymph node metastasis in CC patients, we hypothesized ER-α36 participated in the metastasis of CC cells. The transwell assay exhibited that in the shNC+E_2_ groups, E_2_ enhanced the abilities of migration and invasion of CC cells compared with the shNC groups, but no obvious difference was found between shER-α36+E_2_ groups and shER-α36 groups. Comparison showed that ER-α36 knockdown inhibited the ability of metastasis of CC cells mediated by E_2_ in the shNC+E_2_ and shER-α36+E_2_ groups, while there is no statistical difference between shNC and shER-α36 groups (**Figure 2F**). There was no remarkable difference between si-NC+E_2_ groups and si-ER-α66 +E_2_ groups (**Supplementary Figure 1F**). In conclusion, ER-α36 silence inhibited proliferation and metastasis of CC cells induced by E_2_ *in vitro*.

### Overexpression of ER-α36 Promotes E_2_-Mediated Proliferation and Metastasis of CC Cells *In Vitro*

**Figure 3:**
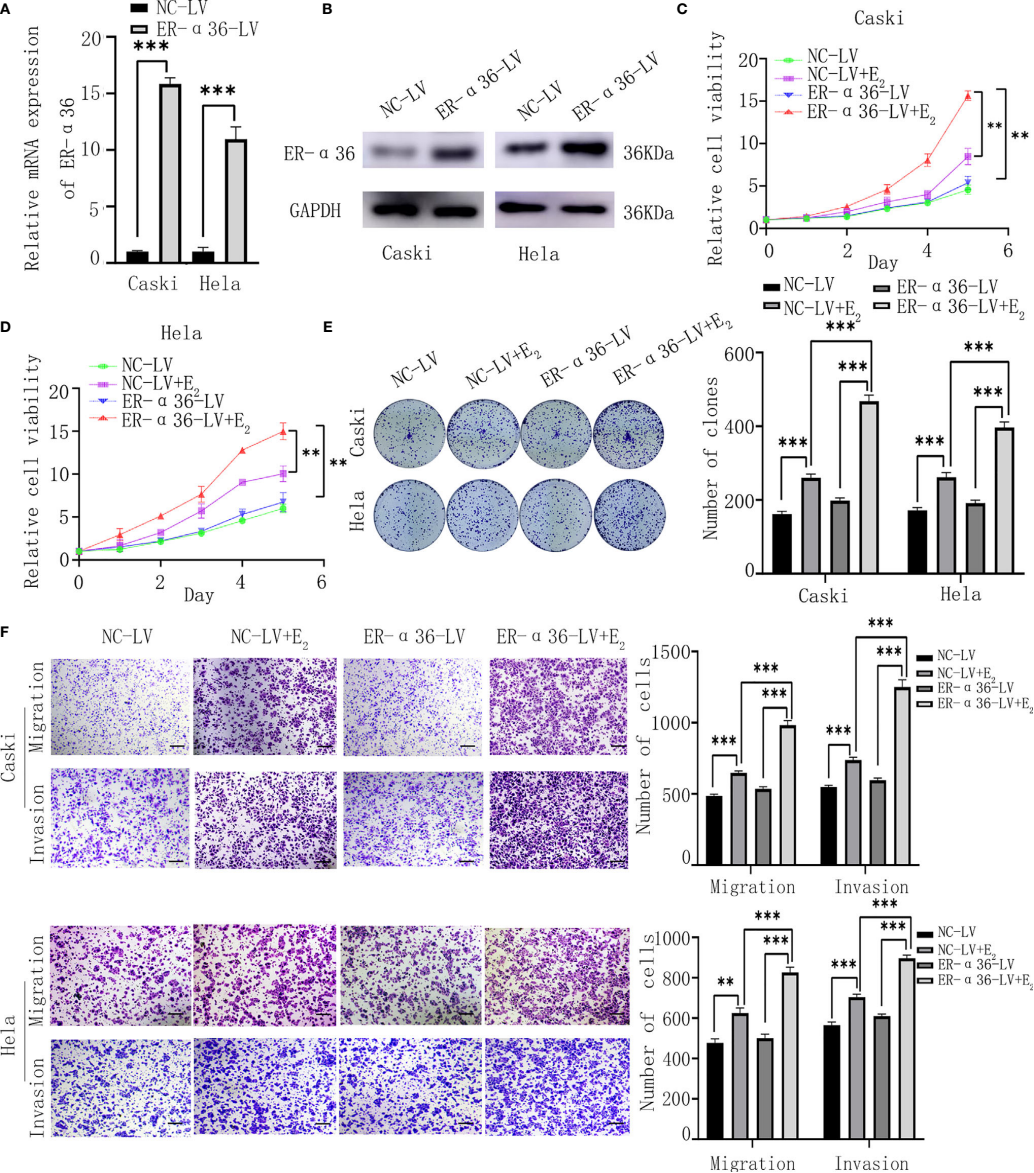
ER-α36 overexpression promotes E_2_-mediated proliferation and metastasis of CC cells in *vitro*. Caski, hela cells were stably transfected with NC-LV, ER-α36-LV and treated with ethanol or 1nM E_2_. **(A, B)** qRT‐PCR and western blotting analysis of the mRNA and protein levels of ER-α36 in transfected caski and hela cells. **(C–E)** Proliferation of caski and hela cells was measured by CCK-8 assay and clonogenic assay after ER-α36 overexpression. **(F)** Migration and invasion of caski and hela cells were evaluated by transwell assay after ER-α36 overexpression. Scale bar: 50μm. (Data are mean ± SEM, **p < 0.01, ***p < 0.001, n = 3).

Caski and hela cells were successfully transfected with ER-α36-LV and NC-LV (**Figures 3A, B**). Cells were divided into 4 groups; the NC-LV groups (cells transfected with NC-LV and treated with ethanol), the NC-LV+E_2_ groups (cells transfected with NC-LV and treated with 1nM E_2_), the ER-α36-LV groups (cells transfected with ER-α36-LV and treated with ethanol) and ER-α36-LV+E_2_ groups (cells transfected with ER-α36-LV and treated with 1nM E_2_). The CCK8 assay showed that cells transfected with ER-α36-LV (ER-α36-LV groups vs. ER-α36-LV+E_2_ groups) displayed higher sensitivity to E_2_ stimulation than the control groups (NC-LV groups vs. NC-LV+E_2_ groups). In the ER-α36-LV+E_2_ groups, the proliferation induced by E_2_ was enhanced in comparison to the NC-LV+E_2_ groups. Nevertheless, there was no remarkable difference in cell viability between NC-LV and ER-α36-LV groups (**Figures 3C, D**). The clonogenic assay obtained the similar results (**Figure 3E**).

Transwell assay showed that E_2_ enhanced the abilities of migration and invasion of CC cells in both groups (ER-α36-LV+E_2_ vs.ER-α36-LV group and NC-LV+E_2_ vs. NC-LV group). Comparison in the NC-LV+E_2_ and ER-α36-LV+E_2_ groups showed that overexpression of ER-α36 promoted the ability of metastasis of CC cells induced by E_2_, while there was no statistical difference between NC-LV and ER-α36-LV groups (**Figure 3F**). In a word, overexpression of ER-α36 promoted E_2_-mediated proliferation and metastasis of CC cells.

### ER-α36 Is Involved in E_2-_Stimulated Cell Cycle Progression

**Figure 4:**
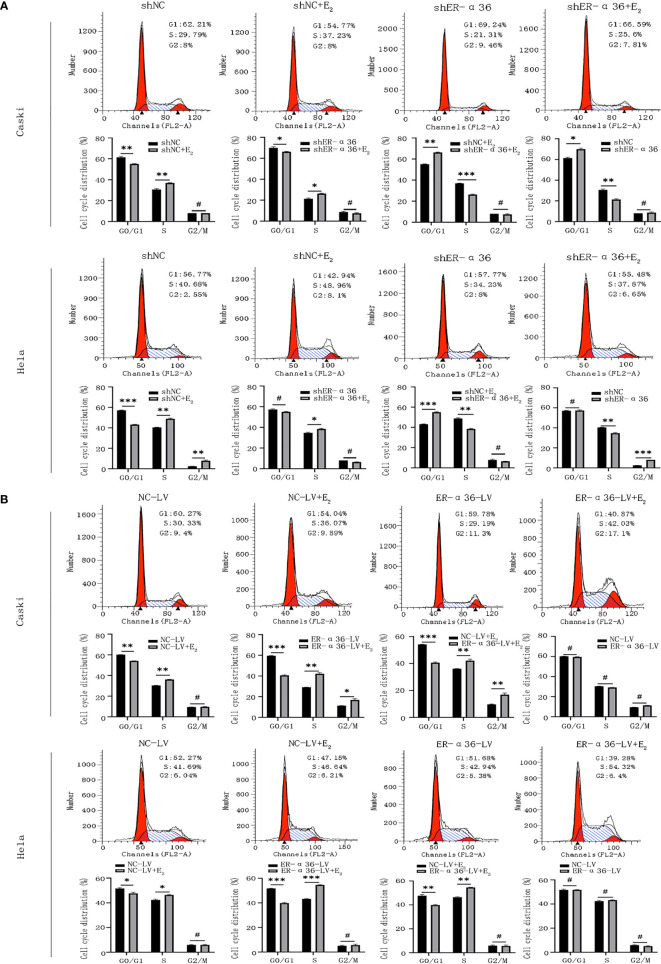
ER-α36 is involved in E_2_ stimulated cell cycle progression. Caski and hela cells transfected with shNC, shER-α36, NC-LV and ER-α36-LV were treated with ethanol or 1nM E_2_ for 24 h in cell cycle assay. **(A, B)** Cell cycle analysis was performed by flow cytometry. Cell cycle phase distribution was expressed as a percentage of total cells as shown. (Data are mean ± SEM, ^#^p > 0.05, *p < 0.05, **p < 0.01, ***p < 0.001, n=3).

Caski and hela cells transfected with shNC, shER-α36, NC-LV, ER-α36-LV, si-NC and si-ER-α66 were incubated with ethanol or 1nM E_2_ for 24 hours. Cell cycle analysis revealed that E_2_ decreased the percentage of cells in G1 phase and increased the percentage of cells in S phase. Comparing shNC+E_2_ groups and shER-α36+E_2_ groups, we found that ER-α36 knockdown increased the percentage of cells in G1 phase and decreased the percentage of cells in S phase, while in ER-α36-LV+E_2_ groups, up-regulated ER-α36 decreased the percentage of cells in G1 phase and increased the percentage of cells in S phase in comparison to the NC-LV+E_2_ groups (**Figures 4A, B**). There was no remarkable difference between si-NC+E_2_ and si-ER-α66+E_2_ groups in cell cycles (**Supplementary Figures 2A, B**). Therefore, these results indicated that ERα36 was involved in the cell cycle progression of CC cells induced by E_2_.

### ER-α36 Enhances the Proliferation of CC Cells Mediated by E_2_ *In Vivo*

**Figure 5:**
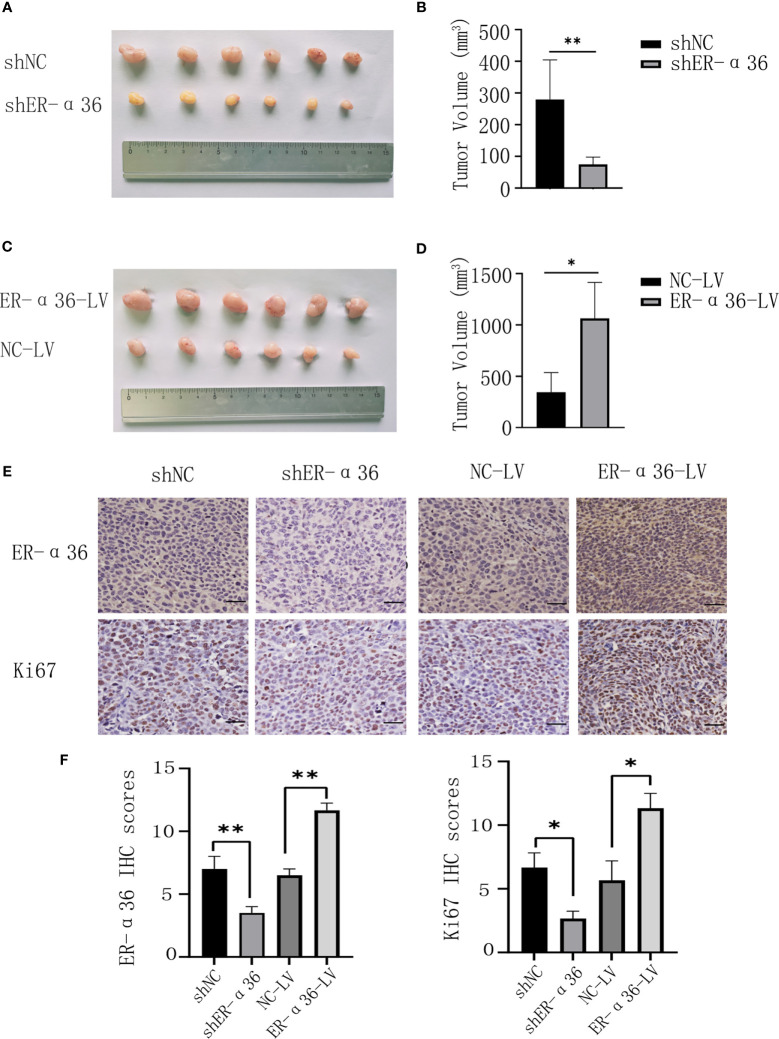
The role of ER-α36 in the oncogenic function induced by E_2_ in *vivo*. **(A, C)** Image of xenograft tumors. **(B, D)** Tumor volumes of each group. **(E)** Representative images of IHC staining of ER-α36 and Ki67 in tumor tissues. **(F)** IHC scores of each group. Scale bar: 50 µm (Data are mean ± SEM, *p < 0.05, **p < 0.01, n = 6).

To further explore the role of ER-α36 in the oncogenic function induced by E_2_ *in vivo*, caski cells transfected with shNC, shER-α36, NC-LV, NC-ER-α36 were subcutaneously injected into the armpits of female nude mice which were subcutaneously inoculated with 0.36 mg 60-day released E_2_ pellets. Consistent with the results *in vitro*, the volumes of the tumors in the shER-α36 group were remarkably decreased compared with those in the shNC groups, and the tumors in the ER-α36-LV groups were boosted as compared to the control group (**Figures 5A–D**). IHC staining was performed to tumor mass, the results showed tumors in shER-α36 groups exhibited significantly lower ER-α36 expression and Ki67 proliferation index vs. the control group, whereas tumors in ER-α36-LV groups exhibited up-regulated ER-α36 expression and high staining intensity of Ki67 (**Figures 5E, F**). These results indicated ER-α36 enhanced the proliferation of CC cells mediated by E_2_ *in vivo*.

### HMGA2 Is a Downstream Target of ER-α36 in CC, Elevated HMGA2 Expression Is Correlated With the Poor Prognosis of CC

**Figure 6:**
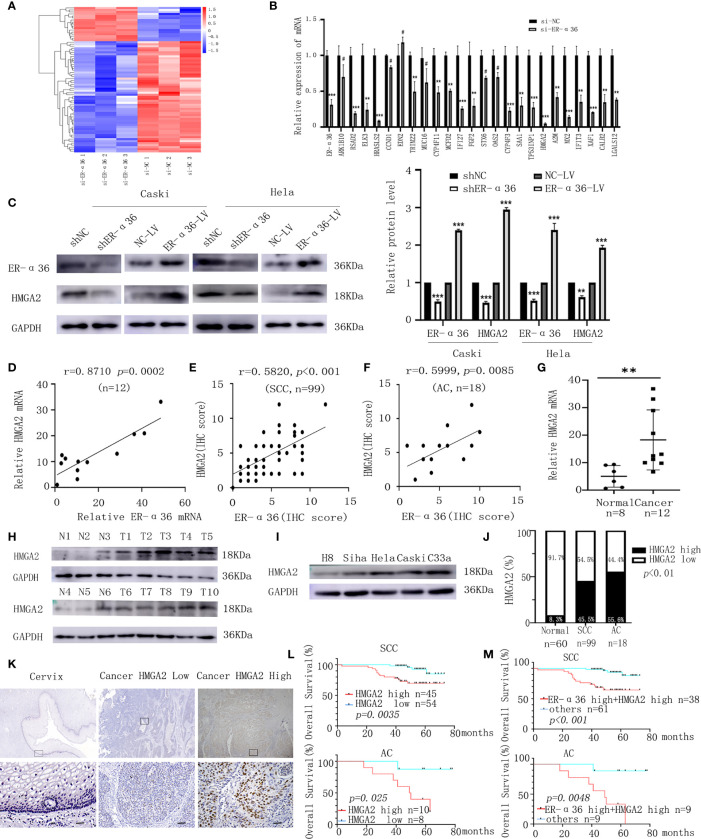
HMGA2 is a downstream target of ER-α36 in CC, elevated HMGA2 predicts unfavorable prognosis in patients with CC. Caski cells transfected with si-ER-α36 and si-NC were treated by 1nM E_2_ for 24 hours in next-generation sequencing (NGS) assay. **(A)** Heatmap of differentially expressed genes profiles in caski cells transfected with si-ER-α36 and si-NC (High and low expression levels are indicated by red and blue, respectively). **(B)** The mRNA levels of top 24 downregulated genes in caski cells with ER-α36 knockdown were detected by qRT-PCR. **(C)** Western blotting analysis of HMGA2 protein expression after ER-α36 knockdown and overexpression. **(D) **Correlation analysis between ER-α36 and HMGA2 expression in fresh-frozen CC tissues. **(E, F)** Association of IHC scores between ER-α36 and HMGA2 in cervical SCC and cervical AC tissues. **(G)** The mRNA level of HMGA2 in normal cervical tissues and CC tissues. **(H)** The protein level of HMGA2 in CC tissues(T1-T10) and normal cervical tissues(N1-N6). **(I)** The protein levels of HMGA2 in H8, siha, hela, caski and C33a cell lines. **(J)** The high and low expression rate of HMGA2 in 60 normal cervical tissues, 99 cervical SCC tissues and 18 cervical AC tissues. **(K)** Representative images of IHC staining of HMGA2 in CC tissues and normal cervical tissues (upper, ×40; lower, ×400, Scale bar: 50 µm). **(L)** Elevated HMGA2 expression correlates with the poor prognosis in cervical SCC patients and cervical AC patients. **(M)** Overall survival curves for cervical SCC patients and cervical AC patients were stratified according to patients with high expression of ER-α36 and HMGA2, and other patients. (^#^p>0.05, **p < 0.01, ***p<0.001).

To illuminate the mechanisms by which ER-α36 promotes CC’s malignant progression induced by E_2_, next-generation sequencing (NGS) was conducted. A total of 72 differentially expressed genes (DEGs, fold change ≥2, p < 0.05) were identified, including 17 upregulated genes and 55 downregulated genes (**Figure 6A**). The top 24 downregulated genes were chosen to validate the DEGs by qRT-PCR assay (**Figure 6B**). HMGA2 was shown to be particularly downregulated and has been confirmed to be associated with tumorigenesis. Therefore, we hypothesized that ER-α36 might promote the malignancy of CC by regulating HMGA2 expression. Western blotting assay showed that the protein level of HMGA2 in CC cells was decreased after ER-α36 knockdown and increased after ER-α36 overexpression (**Figure 6C**). Moreover, qRT-PCR (**Figure 6D**) and IHC (**Figures 6E, F**) assays revealed HMGA_2_ expression was positively correlated with ER-α36 expression in CC tissues. These results suggested that HMGA2 was a downstream effector of ER-α36 in CC.

To explore the role of HMGA2 in CC, we detected the expression of HMGA2 in CC tissues and cervix tissues with qRT-PCR and western blotting, the results showed that the mRNA and protein levels of HMGA2 were remarkably higher in cancer tissues (**Figures 6G, H**). Then we examined HMGA2 expression in H8, siha, hela, caski and C33a cell lines, western blotting revealed HMGA2 was significantly over-expressed in four CC cell lines (siha, hela, caski and C33a) compared with H8 cells (**Figure 6I**). To evaluate the expression pattern and clinical significance of HMGA2 in CC, IHC staining assay was applied. The results verified the expression of HMGA2 was elevated in CC tissues (**Figures 6J, K**). In cervical SCC patients, clinicopathological characteristic analysis showed HMGA2 expression was correlated with FIGO stage (p=0.012), DSI (p=0.03), lymph node metastasis (p=0.005) and ER-α36 expression (p<0.001, **Table 5**). In cervical AC patients, high expression of HMGA2 was associated with positive lymph node metastasis (p=0.036) and elevated ER-α36 expression (p=0.00, **Table 6**). Kaplan-Meier survival curves showed high HMGA2 expression was associated with low overall survival rate in both cervical SCC and cervical AC patients (**Figure 6L**). Furthermore, the expressions of ER-α36 and HMGA2 were combined to evaluate the prognostic value of co-expression of ER-α36 and HMGA2. Kaplan-Meier survival curve revealed patients with high expression of ER-α36 and HMGA2 seemed to have worse prognosis than others (**Figure 6M**), indicating that co-expression of ER-α36 and HMGA2 may be a more sensitive factor in CC. In conclusion, HMGA2 was highly expressed in CC tissues and predicted unfavorable prognosis.

**Table 5:** Association between HMGA2 expression and clinicopathologic characteristics of cervical SCC patients.

Characteristic	Cases	HMGA2 expression		p-value
		Low	High	
Age				0.26
≤50	60	30	30	
>50	39	24	15	
Menopausal status				0.33
Postmenopausal	29	18	11	
Premenopausal	70	36	34	
Differentiation				0.179
Poor	68	34	34	
Well/moderate	31	20	11	
FIGO stage				0.012*
I	81	49	32	
II	18	5	13	
Tumor Size				0.744
<4cm	71	38	33	
≥4cm	28	16	12	
DSI				0.03*
<1/2	9	8	1	
≥1/2	90	46	44	
LNM				0.005**
Negative	67	43	24	
Positive	32	11	21	
LVSI				0.337
Negative	79	45	34	
Positive	20	9	11	
ER-α36 status				<0.001***
Low	51	44	7	
High	48	10	38	

SCC, squamous cell carcinoma; FIGO, International Federation of Gynecology and Obstetrics; DSI, deep stromal invasion; LNM, lymph node metastasis; LVSI, lymphvascular space involvement.

*p < 0.05, **p < 0.01, ***p < 0.001, χ2 test.

**Table 6:** Association between HMGA2 expression and clinicopathologic characteristics of cervical AC patients.

Characteristic	Cases	HMGA2 expression		p-value
		Low	High	
Age				0.608
≤50	13	5	8	
>50	5	3	2	
Menopausal status				0.608
Postmenopausal	5	3	2	
Premenopausal	13	5	8	
Differentiation				1
Poor	12	5	7	
Well/moderate	6	3	3	
FIGO stage				0.216
I	15	8	7	
II	3	0	3	
Tumor Size				0.608
<cm	13	5	8	
≥4cm	5	3	2	
DSI				0.559
<1/2	3	2	1	
≥1/2	15	6	9	
LNM				0.036*
Negative	13	8	5	
Positive	5	0	5	
LVSI				1
Negative	16	7	9	
Positive	2	1	1	
ER-α36 status				0.003**
Low	8	7	1	
High	10	1	9	

AC, adenocarcinoma; FIGO, International Federation of Gynecology and Obstetrics; DSI, deep stromal invasion; LNM, lymph node metastasis; LVSI, lymphvascular space involvement.

*p < 0.05, **p < 0.01, Fisher test.

### HMGA2 Knockdown Impairs Proliferation and Metastasis of CC Cells

**Figure 7:**
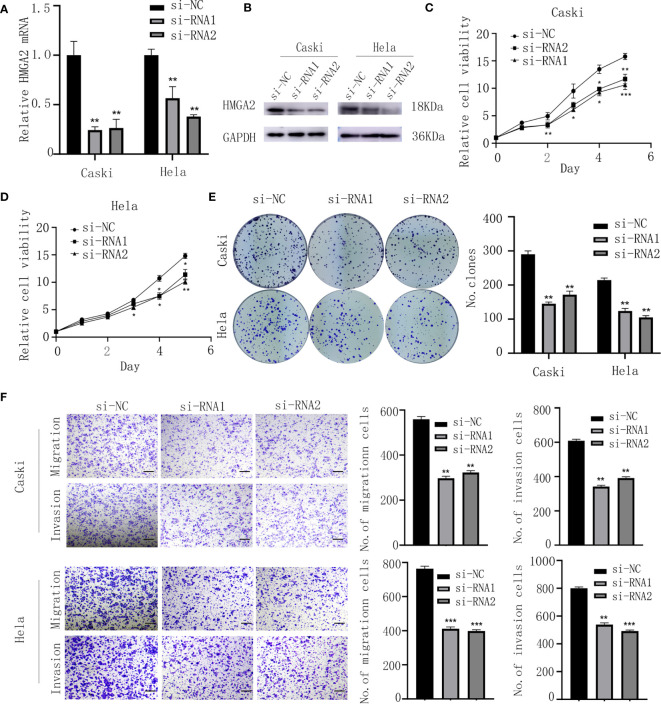
Knockdown of HMGA2 inhibits the proliferation, migration and invasion of CC cells. **(A, B)** Successfully knockdown of HMGA2 using two independent siRNAs in caski and hela cells was verified by qRT-PCR and western blotting. **(C, D)** Proliferation of caski and hela cells was measured by CCK8 assay after HMGA2 knockdown. **(E)** Colony formation efficiency of caski and hela cells was assessed by clonogenic assay after HMGA2 knockdown. **(F)** Migration and invasion of caski and hela cells were evaluated by transwell assay after transfection with si-HMGA2 or si-NC. Scale bar; 50μm. (Data are mean ± SEM, *p < 0.05, **p < 0.01, ***p < 0.001, n = 3).

To assess the biological function of HMGA2 in CC cells, si-HMGA2 and its negative control(si-NC) were transfected into caski and hela cells (**Figures 7A, B**). CCK8 and colony formation assays showed HMGA2 silence inhibited the proliferation of CC cells (**Figures 7C–E**). Transwell assay revealed that HMGA2 knockdown decreased the migration and invasion ability of CC cells (**Figure 7F**). Taken together, these data implied the oncogenic effects of HMGA2 in CC.

### ER-α36 Promotes E_2_-Induced Malignancy of CC by Regulating HMGA2

**Figure 8:**
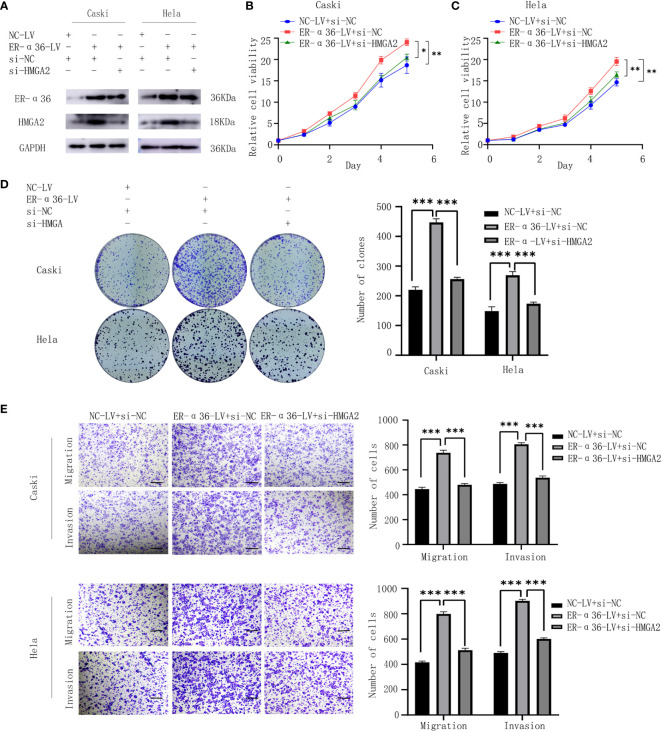
ER-α36 promotes E_2_-induced malignancy of CC depending on HMGA2. si-HMGA2 and si-NC were introduced into caski and hela cells which were previously transfected with NC-LV or ER-α36-LV, Cells were treated with 1nM E_2_. **(A)** Western blotting was used to assess HMGA2 and ER-α36 expression. Cell proliferation was detected by CCK8 assay **(B, C)** and colony formation assay **(D)**. Migration and invasion abilities were examined by transwell assay **(E)**. Scale bar: 50μm. (Data are mean ± SEM, *p < 0.05, **p < 0.01, ***p < 0.001, n = 3).

To verify whether E_2_ and ER-α36 promote aggressive behaviors of CC through HMGA2, a rescue experiment was performed. We introduced si-HMGA2 and si-NC into caski and hela cells that were previously transfected with NC-LV or ER-α36-LV(**Figure 8A**). Cells were treated with 1nM E_2_. The results showed that knockdown of HMGA2 obviously attenuated the proliferation and metastasis induced by ER-α36 overexpression (**Figures 8B–E**). These results suggested that HMGA2 was involved in E_2_ and ER-α36 mediated oncogenic behaviors of CC cells.

## Discussion

Development of CC has generally been considered to be unrelated to estrogen, however, a growing number of research has found that estrogen and its receptor ER-α were not only involved but also played important roles in the genesis and development of cervical carcinoma (9, 10, 23–25). ER-α has three isoforms: ER-α36, ER-α46 and ER-α66. ER-α36 is a truncated variant of ER-α66, with a unique 27 amino acid domain at the C-terminal, which may endow it different characteristics (11). In terms of expression, ER-α66 could suppress ER-α36 expression by inhibiting ER-α36 promoter activity through the AF-1 domain, on the contrary, ER-α36 could also suppress ER-α66 expression by negatively regulating the transcription of ER-α66 (26). Previous studies have found ER-α36 was upregulated in various cancer tissues, such as lung (20), gastric (27), primary hepatocellular (28), thyroid (29) and breast (30) cancer, and correlated with poor prognosis. Consistent with these researches, we found ER-α36 was over-expressed in CC cell lines and tissues, and predicted unfavorable prognosis, while ER-α66 was low-expressed in these cell lines and tissues, and had no prognostic significance in CC. Sun et al. (31) reported that ER-α36 was mainly expressed in the plasma membrane and cytoplasm of caski and hela cells. In our study, we got the similar results. The IHC assay showed that ER-α36 was mainly located in the cellular membrane and cytoplasm of cervical cancer tissues, only a small part of it was located in the nucleus. In addition, according to analyze the correlation between clinical characteristics and ER-α36 expression, we discovered in cervical SCC, elevated ER-α36 was associated with advanced FIGO stage, deeper stromal invasion, positive lymph node metastasis and high expression of Ki67 (the proliferation index), in AC, high expression of ER-α36 was associated with positive lymph node metastasis. These results indicated the oncogenic role of ER-α36 in CC.

Tong et al. (14) reported that ER-α36 could rapidly activate the PKCδ/ERK pathway in response to E_2_, leading to an increase of cyclin D1/cyclin-dependent kinase 4, resulting in the promotion of cell cycle progression and proliferation in endometrial cancer. Chaudhri et al. (17) demonstrated ER-α36 activated MAPK/ERK and PI3K/AKT paths under the stimulation of E_2_, contributing to metastasis of breast cancer. Nofrat Schwartz et al. (21) found that ER-α36 could promote the aggression of laryngeal cancer through PKC pathways induced by E_2_. These studies indicated E_2_ and ER-α36 were involved in the progression of certain malignant tumors. Therefore, we hypothesized that E_2_ and ER-α36 may be related to the development of CC.

In our study, we found E_2_(1nM, the level equivalent to that in premenopausal women (32) promoted the cell cycle progression, and enhanced the proliferation and metastasis of CC cells. Furthermore, we verified it was ER-α36 not ER-α66 that promoted the malignant behaviors of CC cells induced by E_2_.

In order to illuminate the mechanism by which ER-α36 promotes CC’s malignant progression induced by E_2_, we conducted NGS and found the expression of HMGA2 was particularly down-regulated in the si-ER-α36 groups. Then we detected the protein level of HMGA2 in caski and hela cells transfected with shER-α36 or ER-α36-LV and their negative controls. The results showed the protein level of HMGA2 was decreased after ER-α36 knockdown and increased after ER-α36 overexpression. In addition, qRT-PCR and IHC staining assays confirmed a positive correlation between the expression of ER-α36 and HMGA2 in CC. HMGA2 is a non-histone nuclear-binding oncofetal protein, which modulates transcription through promoting conformational changes (33). Previous research found HMGA2 was over expressed in embryonic tissue and in various malignant tumors such as colorectal (34), breast (35), pulmonary (36) and ovary cancer, but was rare detected in normal adult tissues (37). In our study, we found the expression of HMGA2 was higher in CC tissues than in normal cervical samples.

Moreover, in cervical SCC, high expressed HMGA2 was correlated with advanced FIGO stage, positive lymph node metastasis, high expression of ER-α36 and poor prognosis. In cervical AC, elevated HMGA2 was associated with positive lymph node metastasis, high ER-α36 expression and predicted poor prognosis. These results indicated that HMGA2 might play an oncogenic role in tumorigenesis and progression of CC. Wei et al. (38) reported HMGA2 promoted the proliferation, migration and invasion of endometrial cancer. Here, we demonstrated HMGA2 silencing suppressed the malignancy behaviors of CC. In addition, knockdown of HMGA2 in ER-α36-overexpressing cells reversed the effect of ER-α36.

Some limitations of our study must be acknowledged. Firstly, the research sample was relatively small. In the future, large-scale cohort samples are necessary to further evaluate the values of ER-α36 in the diagnosis and prognosis of CC. Secondly, the molecular mechanism of the interaction between ER-α36 and HMGA2 has not been elucidated. Although ER-α36 is a membrane receptor, in our study, the expression of ER-α36 was observed in the nucleus of some cervical cancer cells. Wang et al. (30) reported that in ER-α36+ breast cancer cells, tamoxifen or estrogen could induce the nuclear translocation of ER-α36 to regulate the transcriptional activity of ER-α to increase ALDH1A1 expression. Similarly, we speculate that in cervical cancer cells, estrogen promotes nuclear translocation of ER-α36, thereby directly regulating the transcription of HMGA2. In the future, co-immunoprecipitation, dual-luciferase reporter assay and other experiments are needed to reveal the interaction between ER-α36 and HMGA2.

In summary, our study found ER-α36 was highly expressed in CC samples and CC cell lines. Elevated expression of ER-α36 was associated with a poor prognosis in CC. Overexpression of ER-α36 promotes E_2_-mediated proliferation and metastasis. Depletion of ER-α36 caused G0/G1 arrest, decreased the sensitivity of CC cells to E_2_. We further demonstrated that ER-α36 promoted E_2_-induced malignancy of CC by regulating HMGA2 expression. In conclusion, ER-α36 might be a novel therapeutic target for the treatment of CC.

## Data Availability Statement

The datasets presented in this study can be found in online repositories. The names of the repository/repositories and accession number(s) can be found below: https://www.ncbi.nlm.nih.gov/, GSE173120.

## Ethics Statement

The studies involving human participants were reviewed and approved by Ethics Committee of Shandong University QIlu Hospital. The patients/participants provided their written informed consent to participate in this study. The animal study was reviewed and approved by Ethics Committee of Shandong University Cheeloo College of Medicine.

## Author Contributions

XY, QS, and CW designed this study. TZ, SZ, and KW collected samples and information of CC patients. CW performed the experiments, analyzed the data and wrote the manuscript. XY revised the manuscript. All authors contributed to the article and approved the submitted version.

## Funding

This work was financially supported by National Natural Science Foundation of China (no. 81874105).

## Conflict of Interest

The authors declare that the research was conducted in the absence of any commercial or financial relationships that could be construed as a potential conflict of interest.
