# Supplementary material for: ER-α36 Promotes the Malignant Progression of Cervical Cancer Mediated by Estrogen via HMGA2
Source: Front Oncol. 2021 Jul 14;11:712849. doi: 10.3389/fonc.2021.712849 (PMC8317436; doi:10.3389/fonc.2021.712849)
Supplement: Supplementary file 1 [file DataSheet_1.doc]

Supplementary Material

**1 Supplemental Table S1 Primers for qRT-PCR**

| Genes | Forward primer(5’-3’) | Reverse primer(5’-3’) |
| --- | --- | --- |
| GAPDH | GTCTCCTCTGACTTCAACAGCG | ACCACCCTGTTGCTGTAGCCAA |
| ER-α36 | GAGAATCCTGAACTTGCATCCT | AAAATGTCCCCACGTCCACA |
| ER-α66 | TCCAGCACCCTGAAGTCTCT | AGATGCTCCATGCCTTTGTT |
| CYP4F11 | GCTTCTGAAGGACCGTGAACCT | CAAAGTCCTGCGTGCAACATCG |
| AKR1B10 | GAGGACCTGTTCATCGTCAGCA | CGTCCAGATAGCTCAGCTTCAG |
| RSAD2 | CCAGTGCAACTACAAATGCGGC | CGGTCTTGAAGAAATGGCTCTCC |
| ELK3 | TTCTGACTCCGAGTCCACTGCT | AGCTCTGTCCAGACTGGGGATT |
| MCFD2 | TCGCCACAAGAATTGCAGCTCC | CACTCATTAGTGGTGCCTGTTCA |
| HRASLS2 | TGGCTATGTGGTCCATCTGGCT | CACCACAGACAGCAGTTCCTTC |
| CCND1 | TCTACACCGACAACTCCATCCG | TCTGGCATTTTGGAGAGGAAGTG |
| EDN2 | CGTGTTCCAGACTGGCAAGACA | CCTCCTGTTGTCGCTTGGCAAA |
| IFI27 | CGTCCTCCATAGCAGCCAAGAT | ACCCAATGGAGCCCAGGATGAA |
| TRIM22 | GGATCGTCAGTAGAGATGCTGC | GAACTTGCAGCATCCCACTCAG |
| MUC16 | GATGTCAAGCCAGGCAGCACAA | GAGAGTGGTAGACATTTCTGGGC |
| FGF2 | AGCGGCTGTACTGCAAAAACGG | CCTTTGATAGACACAACTCCTCTC |
| OAS2 | GCTTCCGACAATCAACAGCCAAG | CTTGACGATTTTGTGCCGCTCG |
| CYP4F3 | CCACCTACATCAAGCCTGTGCT | GGCTCCACTTTTCACCAGCACT |
| SAA1 | TCGTTCCTTGGCGAGGCTTTTG | AGGTCCCCTTTTGGCAGCATCA |
| TP53INP1 | TGATGAATGGATTCTTGTTGACTTC | TGAAGGGTGCTCAGTAGGTGAC |
| XAF1 | CCTCCATGAGGCTTACTGCCTG | GAAACTCCAGCGAGGACTTCTG |
| CALB2 | GATCCTGCCAACCGAAGAGAAC | CGATGTAGCCACTCCTGTCTGT |
| IFIT3 | CCTGGAATGCTTACGGCAAGCT | GAGCATCTGAGAGTCTGCCCAA |
| A2M | GTTGAAGAGCCTCACACGGAGA | TTCCACTCGGTGATGGTGTCAG |
| MX2 | AAAAGCAGCCCTGTGAGGCATG | GTGATCTCCAGGCTGATGAGCT |
| LGALS12 | GATGGTCATGCTGCAAGGAGTG | TGGTGGTATGGAAGCGAGGGTT |
| HMGA2 | GAAGCCACTGGAGAAAAACGGC | GGCAGACTCTTGTGAGGATGTC |
| STX6 | CACGAATTGGAGAGCACTCAGTC | GAGGATGAGCACAACCAACAGG |

**2 siRNA sequence**

The siRNA sequences for si-ER-α66 and si-NC were as follows:

si-RNA1:5’-GGUCCACCUUCUAGAAUGUTT-3’

5’-ACAUUCUAGAAGGUGGACCTT-3’

si-RNA2:5’-CAGGCCAAAUUCAGAUAAUTT-3’

5’-AUUAUCUGAAUUUGGCCUGTT-3’

si-NC: 5’-UUCUCCGAACGUGUCACGUTT-3’

5’-ACGUGACACGUUCGGAGAATT-3’

The siRNA sequences for si-ER-α36 and si-NC were as follows:

si-ER-α36:5’-CUGGCUAGAGAUCCUGAUGAU-3’

5’-AUCAUCAGGAUCUCUAGCCAG-3’

si-NC: 5’-UUCUCCGAACGUGUCACGUTT-3’

5’-ACGUGACACGUUCGGAGAATT-3’

The siRNA sequences for si-HMGA2 and si-NC were as follows:

si-RNA1:5’-GGCCACAACAAGUUGUUCATT-3’

5’-UGAACAACUUGUUGUGGCCT-3’

si-RNA2:5’-GGACAAUCUACUACCAAGATT-3’

5’-UCUUGGUAGUAGAUUGUCCTT-3’

si-NC: 5’-UUCUCCGAACGUGUCACGUTT-3’

5’-ACGUGACACGUUCGGAGAATT-3’

**3 Supplemental Figures**

**
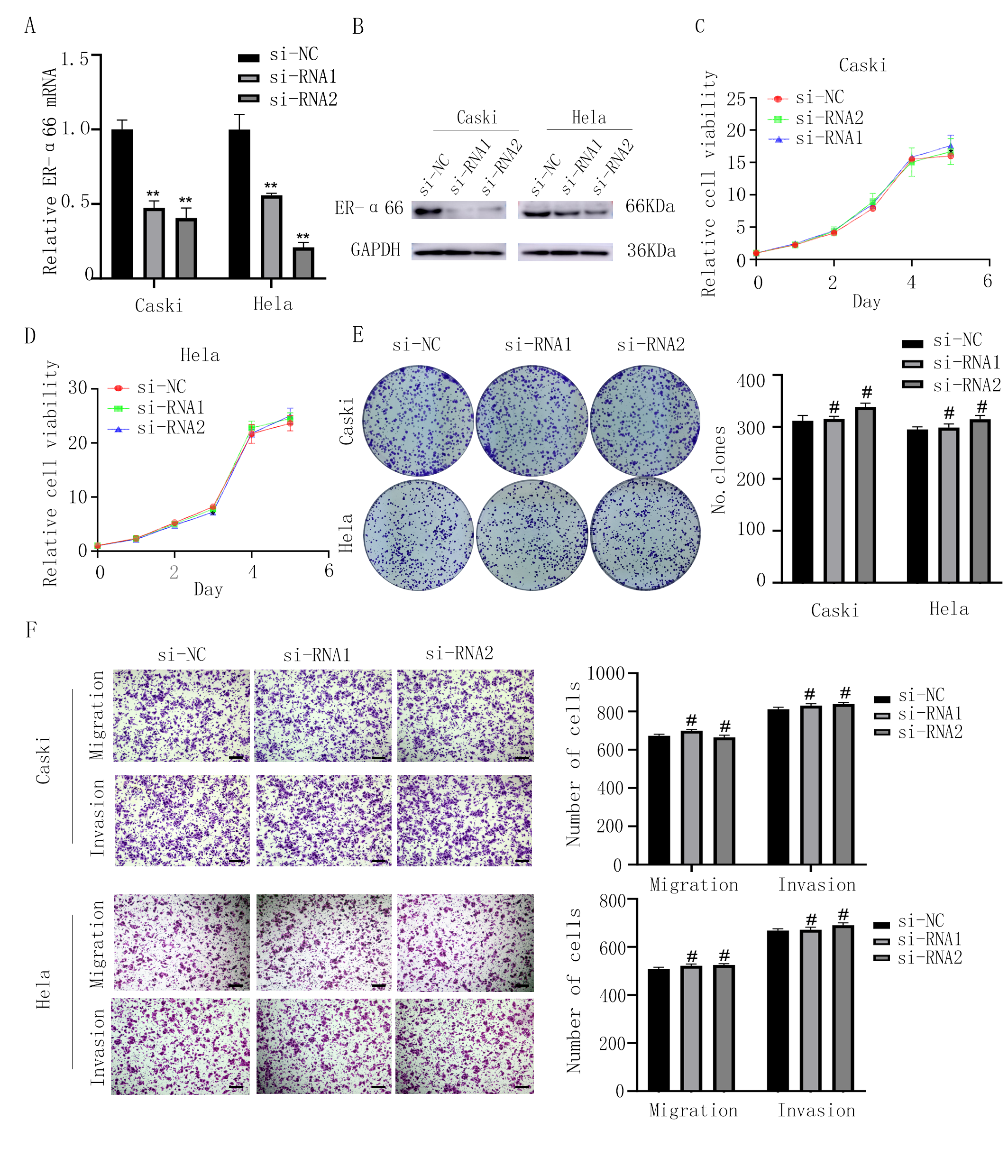
**

**Supplemental figure 1.** The effect of ER-α66 on E_2_-induced proliferation, migration and invasion of cervical cancer cells. Two sequences of siRNA targeting ER-α66 and their negative control (si-NC) were transfected into caski and hela cells, and these cells were treated with 1nM E_2_ in CCK8, colony formation and transwell assays.**(A)** and **(B)** qRT-PCR and western blotting detected ER-α66 expression in caski and hela cells after ER-α66 knockdown. Proliferation of caski and hela cells was measured by CCK-8 assays**(C,D)** and clonogenic assay**(E)** after ER-α66 knockdown. **(F)**Migration and invasion of caski and Hela cells were evaluated by transwell assay after transfection with si-ER-α66 or si-NC. Scale bar: 50μm.(Data are mean ± SEM, ^#^p>o.o5, n = 3).

**Supplemental figure 2.**

**
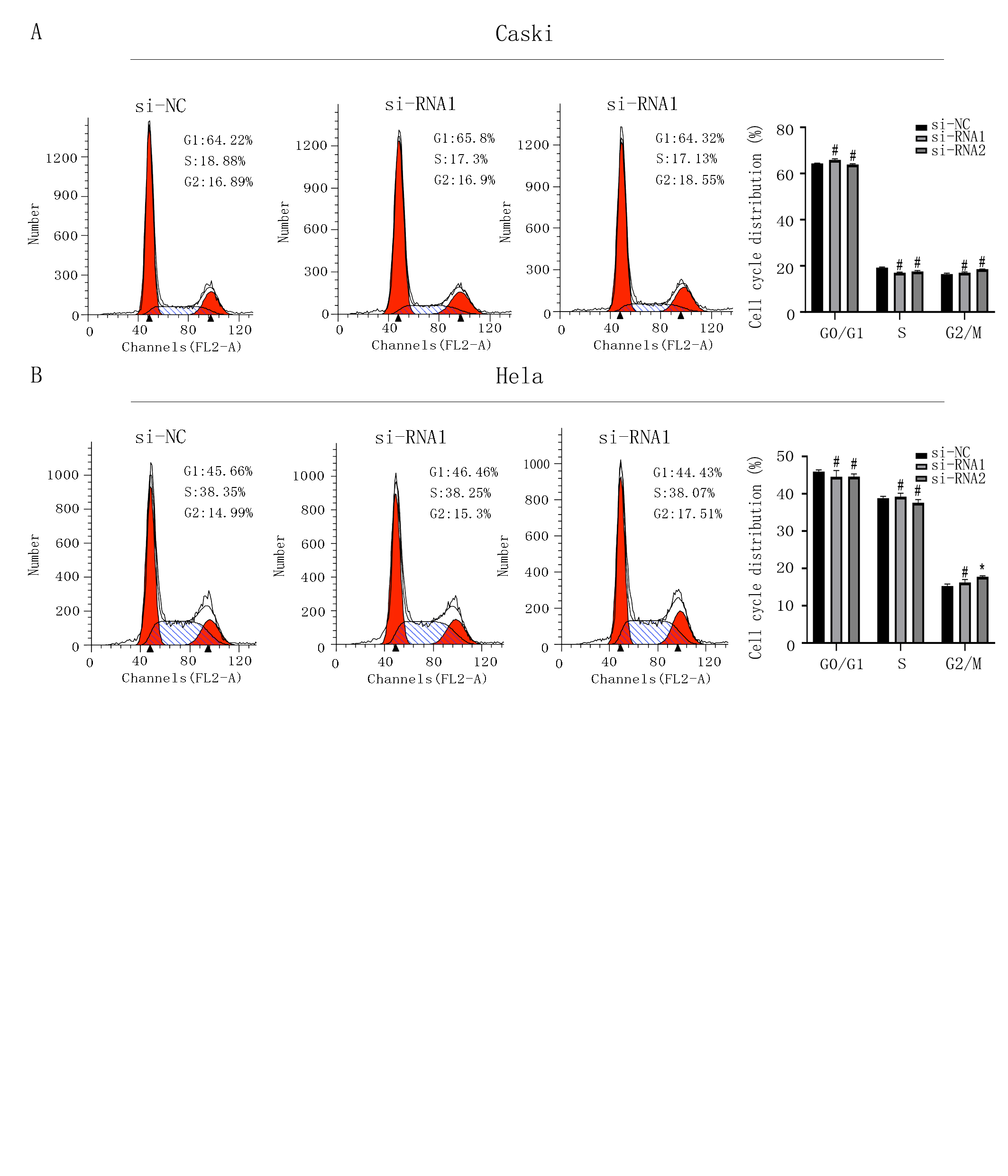
**

**Supplemental figure 2.** The role of ER-α66 on E_2_-induced cell cycle alternation. Si-ER-α66 and si-NC were transfected into caski and hela cells, and these cells were treated with 1nM E_2_ for 24 hours in cell cycle assay. **(A)** and **(B)** Cell cycle distributions of si-ER-α66 and si-NC groups were identified by flow cytometry. Cell cycle phase distribution was expressed as a percentage of total cells as shown. (^#^P>0.05, *P<0.05, n = 3).
